# The Initial Oxidation of HfNiSn Half-Heusler Alloy by Oxygen and Water Vapor

**DOI:** 10.3390/ma14143942

**Published:** 2021-07-14

**Authors:** Oshrat Appel, Shai Cohen, Ofer Beeri, Yaniv Gelbstein, Shimon Zalkind

**Affiliations:** 1 Nuclear Research Centre-Negev, P.O. Box 9001, Beer-Sheva 84190, Israel; oshratap@nrcn.gov.il (O.A.); scking1@gmail.com (S.C.); ofer.beeri@gmail.com (O.B.); 2 Department of Materials Engineering, Ben-Gurion University of the Negev, P.O. Box 653, Beer-Sheva 84105, Israel; yanivge@bgu.ac.il

**Keywords:** HfNiSn, half-Heusler, thermoelectric, segregation, surface oxidation, oxygen, water vapor, XPS

## Abstract

The MNiSn (M = Ti, Zr, Hf) *n*-type semiconductor half-Heusler alloys are leading candidates for the use as highly efficient waste heat recovery devices at elevated temperatures. For practical applications, it is crucial to consider also the environmental stability of the alloys at working conditions, and therefore it is required to characterize and understand their oxidation behavior. This work is focused on studying the surface composition and the initial oxidation of HfNiSn alloy by oxygen and water vapor at room temperature and at 1000 K by utilizing X-ray photoelectron spectroscopy. During heating in vacuum, Sn segregated to the surface, creating a sub-nanometer overlayer. Exposing the surface to both oxygen and water vapor resulted mainly in Hf oxidation to HfO_2_ and only minor oxidation of Sn, in accordance with the oxide formation enthalpy of the components. The alloy was more susceptible to oxidation by water vapor compared to oxygen. Long exposure of HfNiSn and ZrNiSn samples to moderate water vapor pressure and temperature, during system bakeout, resulted also in a formation of a thin SnO_2_ overlayer. Some comparison to the oxidation of TiNiSn and ZrNiSn, previously reported, is given.

## 1. Introduction

The interest in highly efficient thermoelectric materials is constantly increasing, due to the worldwide tendency to reduce greenhouse-gas emissions and global warming and also to focus on energy harvesting for generating electric power. Half-Heusler (HH) alloys are one of the most investigated material systems presently for high temperature thermoelectric energy conversion [1,2] due to their high thermoelectric properties and thermal stability. The most investigated HH alloy is the *n*-type MNiSn-based family, where M stands for Ti, Zr, or/and Hf, which are widely available, relatively low in cost, and nontoxic. By carefully manipulating the alloy composition and structure, the thermoelectric properties can be optimized and reach high efficiency, which makes them a promising candidate for high operating temperatures [3,4,5].

For practical applications, it is crucial to consider also the environmental stability of the compounds at working conditions, which is a key factor for the device’s life span, reliability, and commercial utilization. Nevertheless, only few studies can be found on the HH corrosion behavior, especially at high operating temperatures [6,7,8]. Recently, Kang et al. [9] demonstrated that MNiSn HH alloys can have relatively high oxidation resistivity in air at the operation temperature, and they correlated this oxidation resistivity to the formation of Ni–Sn intermetallic protective layers. Nevertheless, their data at 873 K and after 72 h indicate the formation of an oxide layer, i.e., a few tens micrometer thick, with most of the oxide phases found to be SnO_2_, HfO_2_ and ZrO_2_.

As part of the task to map the surface properties and the oxidation of the MNiSn family, we previously characterized for the first time the surface of the TiNiSn and ZrNiSn alloys and their initial interaction with oxygen and water vapor at room temperature (RT) and 1000 K, using surface sensitive techniques such as Auger electron spectroscopy (AES) and X-ray photoelectron spectroscopy (XPS) [10,11]. It was found that during heating the samples in vacuum, Sn readily segregates to the surface; this was attributed to the considerably lower surface energy of the Sn compared to the other elements in the alloy. The oxidation of the alloys was governed by the propensity of Ti and Zr to oxidize, and oxygen-induced segregation of those components to the surface was also observed. The oxidation tendency and oxide phase formation were in accordance with the enthalpy of formation of the component’s oxides.

In the present work, temperature effects on the surface composition and the initial oxidation of HfNiSn alloy by oxygen and water vapor at room temperature (RT) and 1000 K were studied, utilizing X-ray photoelectron spectroscopy (XPS). Some comparisons to the TiNiSn and ZrNiSn alloys’ behavior, presented in the preceding studies [9,10], are given. Understanding the mechanisms that govern the surface composition change and oxidation of these relatively simple ternary alloys, that are presented here, can serve as building blocks to understand the oxidation behavior of more complex HH alloys.

## 2. Experimental Section

The HfNiSn alloy was prepared from high purity components by arc melting, under Ar atmosphere. The alloy was re-melted 5 times, and the composition and homogeneity were verified by scanning electron microscopy (SEM, JSM 5600, JEOL Ltd., Tokyo, Japan) and energy dispersive spectroscopy (EDS) (Thermo Fisher Scientific, Waltham, MA, USA). The crystallographic structure ($F\bar{4}3m$) was verified by X-Ray diffraction (Rigaku DMAX 2100, Tokyo, Japan).

A ~6 mm diameter and 1 mm thick sample was cut out from the casting and was gradually polished using abrasive papers and diamond paste down to 1 µm. The sample was attached to two Ta wires, which enabled heating by driving an electric current through it. The sample’s temperature was monitored by a chromel–alumel thermocouple, attached to the sample edge.

The experiments were performed in an ultrahigh vacuum (UHV) system (ESCALAB 250 (Thermo Scientific, Waltham, MA, USA), pumped to a base pressure of ~2 × 10^−10^ Torr. The vacuum was monitored by a quadrupole residual gas analyzer (RGA) (SRS 100, Stanford Research Systems, Sunnyvale, CA, USA). Hydrogen and some CO traces were found to be the main residual gases. The system contains standard surface analysis instrumentation for XPS and a differentially pumped rastered Ar^+^ gun for surface cleaning. The XPS measurements were performed using an Al-anode x-ray source (hν = 1486.6 eV). The survey scans and the high-resolution Ni 2p peaks were measured with a spectrometer pass energy of 50 eV, while the Hf 4f peaks and Sn 3d were measured with 20 eV pass energy. The Au 4f_7/2_ peak at binding energy (BE) of 84 ± 0.1 eV and Sn 3d_5/2_ at 485 ± 0.1 eV, taken from clean pure reference samples at similar parameters as those of the alloy, were used to verify the energy calibration of the spectrometer.

The oxidation experiments were performed by backfilling the vacuum chamber with O_2_ (99.999%) or water vapor via leak valves to a pressure of up to 1 × 10^−6^ Torr (later on the exposure is given in Langmuir, 1 L = 1 × 10^−6^ Torr × 1 s). This pressure range was selected since it allows the study of the initial oxidation stage under clean UHV conditions. The experiments were performed at RT and 1000 K, which is the upper limit of the use of HH alloys; this allows the comparison to TiNiSn and HfNiSn alloys, as previously studied [9,10].

The spectra were analyzed using the CasaXPS 2.3.22 software (Teignmouth, UK). The XPS data analysis of the high resolution spectra was performed using Shirley background, and the constraints on energy locations of the different oxidation states and the split-orbit doublet ratios were detailed in [12,13,14,15,16,17,18,19]. A mixture of Gaussian–Lorentzian line shape GL (30) was used for the oxides fitting while for the metallic core lines a slightly asymmetric shape line in the form of LA (α,β,m) was used.

## 3. Results and Discussion

### 3.1. Surface Characterization and Segregation

**Figure 1:**
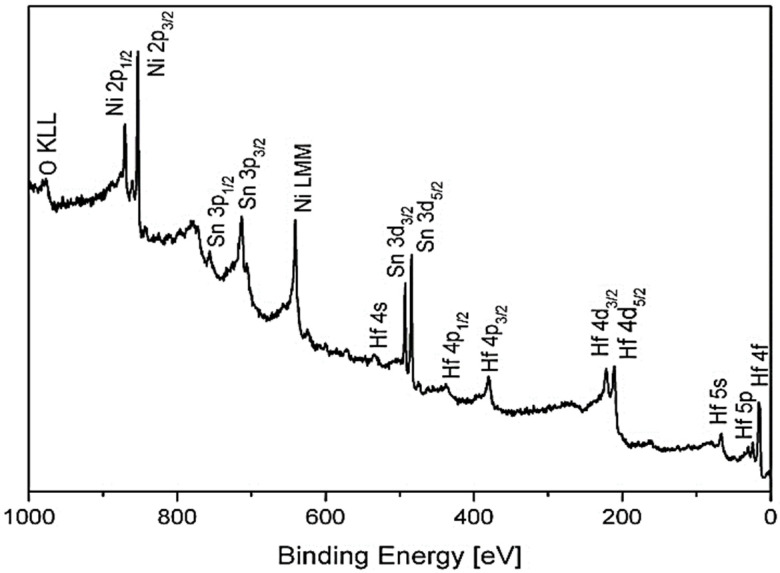
Survey spectrum taken from the HfNiSn alloy surface after sputter-cleaning.

The survey XPS spectra of the HfNiSn after sputter-cleaning at RT are presented in Figure 1. No measurable carbon contamination could be observed, although some small traces of oxygen could be observed from the O KLL signal (there is some overlapping between the O 1s and Hf 4s) even after prolong sputtering. As seen later in Section 3.2, these oxygen traces can be correlated mainly to Hf oxide dispersions, which were formed during the alloy preparation.

In the half-Heusler alloys, which have a general XYZ formula, the X and Y atoms are characterized by a cationic behavior, and the Z atom displays an anionic one. In the MNiSn family, where M denotes Ti, Zr or Hf, these elements and Ni are the cations, transferring a charge towards the anionic Sn. This charge transfer tendency is expressed as chemical shifts of the elements in the alloy, compared to the known BE of the pure elements, as previously reported for the TiNiSn and ZrNiSn [10,11]. Table 1 depicts the measured BE of the elements from the HfNiSn alloy. The values of the BE of the TiNiSn, ZrNiSn, and the known common values of the pure elements from the literature were added for comparison. As can be seen, the Hf, Ti, Zr, and Ni are shifted to higher BE by 0.1–0.3 eV compared to the pure elements, while Sn is shifted to lower BE, in corresponding to the expected cationic and anionic behavior of the elements in the alloy.

**Figure 2:**
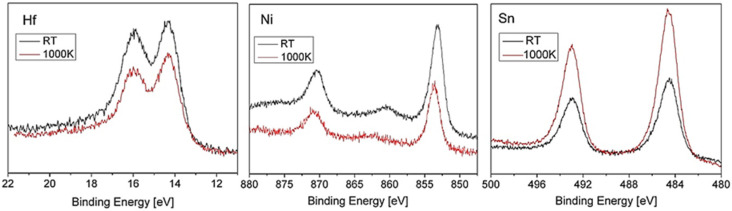
Hf, Ni, and Sn spectra, taken from the alloy before (black) and after (red) heating to 1000 K for 15 min.

During our previous work on TiNiSn and ZrNiSn alloys, it was found that heating the alloys in vacuum causes Sn to segregate to the surface driven by the considerably lower surface energy of Sn compared to the other components [10,11]. Since the surface energy of Sn (~0.7 J/m^2^) is considerably lower than the surface energy of Hf (~2.2 J/m^2^) and Ni (~2.4 J/m^2^) [20], it is expected that Sn would segregate to the surface also in the present case. Figure 2 shows the Hf, Ni, and Sn spectra, taken from the alloy before and after heating to 1000 K for 15 min. The attenuation of the Hf and Ni peaks and the growth of the Sn one indicates that Sn segregates to the surface, as compared to the results monitored in the previous work [10,11]. In addition, the binding energy of the Sn after segregation increases by ~0.1–0.2 eV, indicating the more “metallic” nature of the Sn overlayer compared to the alloy. Assuming that the Sn enriches the surface as a homogenous overlayer, its effective thickness can be evaluated from the intensity attenuation of the underlying elements, according to Equation (1) [21]:

*d* = −λcos *θ* × ln(*I*/*I*_0_) (1)

where *d* is the overlayer thickness, λ is the inelastic mean free path (IMFP) of the electrons passing through the overlayer, and it is commonly evaluated by using the NIST database [22]. *I* is the intensity of the underlying attenuated signal, and *θ* is the angle between the analyzer entrance and the normal to surface.

The IMFP of Hf 4f (E_k_ = 1470 eV) and Ni 2p (E_k_ = 633 eV) in Sn were evaluated as 2.3 nm and 1.2 nm (from the predictive formula by Gries [22]) and the Sn segregated overlayer thickness was evaluated as ≈0.5–0.7 nm, similar to the results obtained in [10,11].

### 3.2. The Interaction with O_2_ at Room Temperature

**Figure 3:**
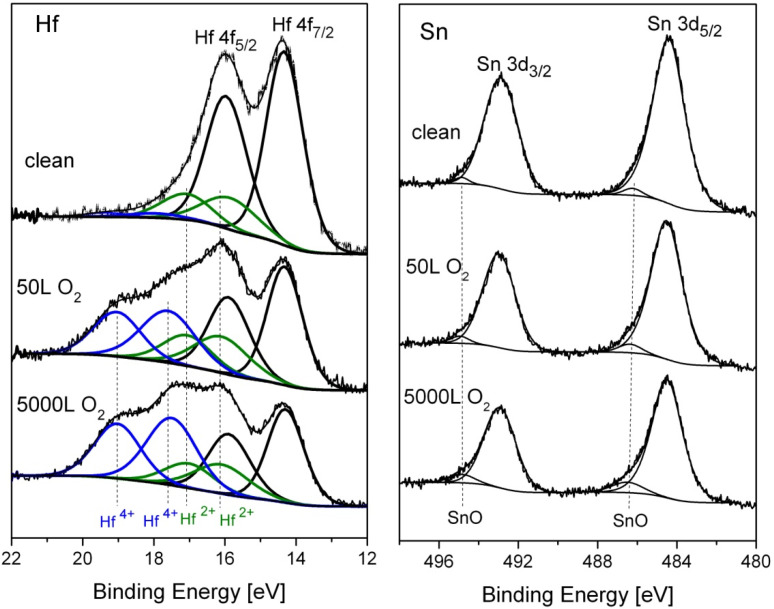
Representative XPS Hf 4f and Sn 3d spectra, after exposing to oxygen at RT, with fitted metallic and oxide components.

XPS spectra of the alloy’s components were recorded after a sequence of oxygen exposure at 1 × 10^−6^ Torr. Representative Hf 4f and Sn 3d spectra with the fitted metallic and oxidic components are depicted in Figure 3. In XPS spectra, all core level signals with quantum number *l* ≥ 1 have a form of a spin-split doublet, with the respective theoretical ratios being 4:3 for f_7/2_ and f_5/2_ and 3:2 for d_5/2_ and d_3/2_. In order to separate the different chemical states constructing the spectra, theoretical peak models representing the chemical states have to be fitted. Trying to fit the clean spectra with only a metallic peak model failed to give the necessary 4:3 ratio between the Hf 4f_7/2_ and Hf 4f_5/2_ levels. It was only after doublets representing Hf^2+^ (HfO) and some Hf^4+^ (HfO_2_) were added to the fitting that very good fits to the data were obtained, with the correct spin-orbit doublet ratios, as can be seen in the figure. In a previous study on Ti_1−x_Hf_x_NiSn alloys [4], it was reported that Hf forms small amounts of Hf oxides dispersion in the alloy grains, and therefore we correlated most of the oxygen traces found to such oxides, which were formed during the alloy preparation (and not to oxygen contamination during the measurements). Exposing the clean surface to oxygen causes mainly an increase in the HfO_2_ while the metallic Hf peaks decrease. In contrast to Hf, only minor oxidation of Sn can be seen, especially at higher exposures, as depicted in Figure 3. No sign for Ni oxidation can be observed at all (spectra not shown, for similar results see Ref. [10]), showing only a reduction in the peak intensity with the increase of oxygen exposure. Since the enthalpy of oxide formation for hafnium (Hf + O_2_ → HfO_2_, ∆H = −268 kcal/mol) is considerably more negative than the oxide formation of Sn (−68 kcal/mol) and Ni (−57 kcal/mol) [23], it is expected from thermodynamic considerations that oxidation progresses mostly by hafnium oxidation.

**Figure 4:**
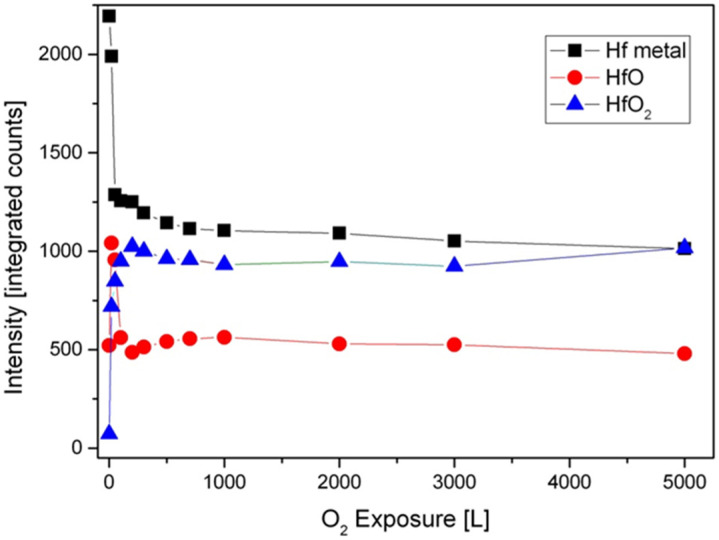
The metallic and oxides intensities of Hf vs. oxygen exposure at RT.

The metallic and oxidic fractions, as extracted from the spectra, are shown in Figure 4. It can be seen that the Hf component in the alloy is readily oxidized at RT, and Hf^4+^ is the principal oxidation state. A small fraction of the oxide can be attributed to a suboxide in the form of HfO at the oxide–metal interface. The amount of this suboxide is increased during the initial oxidation, reaching its maximum intensity at ~20 L, and then its signal attenuates by the growing of the HfO_2_ overlayer.

**Figure 5:**
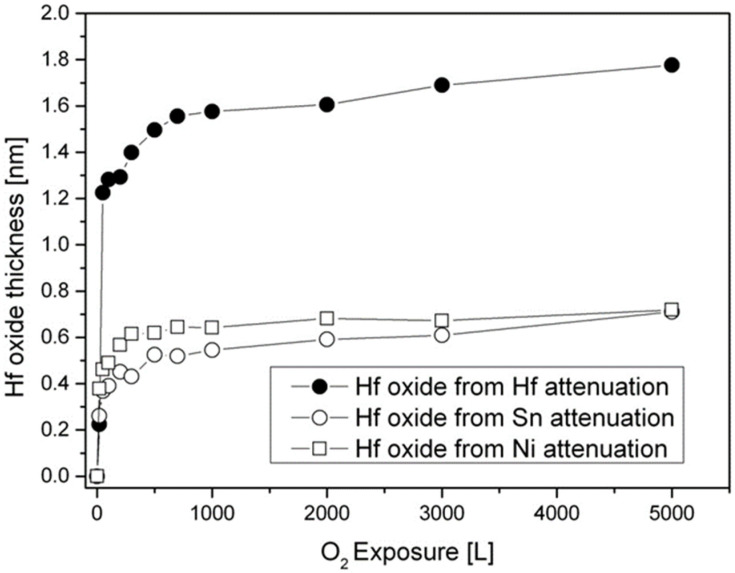
HfO_2_ thickness formed on the surface during oxygen exposure at RT, as calculated from the attenuation of the metallic Hf, Ni, and Sn signals.

Following the intensity of the Sn and Ni lines, it can be seen that they are attenuated during oxidation. Since there is no oxidation of Ni and only slight Sn oxidation, this attenuation implies the possibility that part of the oxidation progresses by some oxygen- induced segregation of Hf to the surface that screens the Sn and Ni signals (as later demonstrated at higher temperatures oxidation). The HfO_2_ thickness formed at RT, as shown in Figure 5, can be estimated from the attenuation of the Hf, Ni, and Sn peaks, using Equation (1), with IMFP of the Ni 2p_3/2_, Sn 3d_5/2_, and Hf 3d_5/2_ electrons passing through the HfO_2_ film evaluated as λ_Ni_ ≈ 1.2 nm, λ_Sn_ ≈ 1.7 nm, and λ_Hf_ ≈ 2.3 nm [22]. It should be emphasized that the evaluation of λ, based on different models, is probably the largest source for uncertainly in the thickness calculations and can differ quite significantly between different models and sources. It can be seen that the oxide thickness obtained from the attenuation of Ni and Sn is about the same, but it give a thinner oxide (0.7 nm at 5000 L O_2_) compared to the thickness evaluated from the Hf line (~1.8 nm). These differences can point to the mechanism of the oxide formation. Since the metallic Hf attenuation is influenced by the total thickness of the formed Hf oxide, it can be deduced that the attenuation of the Ni and Sn peaks are mostly governed by the fraction of the HfO_2_ formed on top of the alloy. Therefore, it can be argued that part of the oxidation occurs by migration of Hf to the surface to react with the oxygen (results in ~0.7 nm oxide) and by oxygen incorporation in the alloy and reacting with Hf to form the other ~1.1 nm oxide.

### 3.3. The Interaction with O_2_ at 1000 K

**Figure 6:**
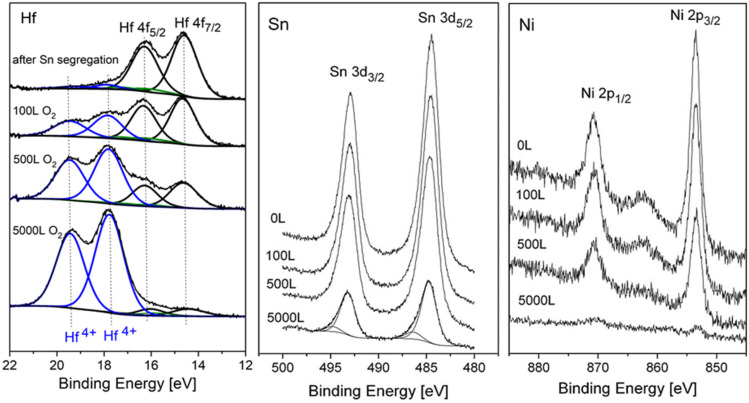
Representative XPS spectra of Hf, Sn and Ni after exposing to oxygen at 1000 K.

Prior to the oxygen exposure at the elevated temperature, the sample was heated in vacuum at 1000 K for 15 min, to allow for Sn segregation to the surface. After each dose, the oxygen was pumped out and the sample was cooled down to RT prior to XPS measurement (to prevent oxide dissolution during the measurements). The representative XPS spectra of the alloy components during oxidation are presented in Figure 6. The HF spectra show rapid oxidation, an almost disappearance of the metallic Hf signal at higher exposures, and the formation of mainly the Hf^4+^ oxidation state. In contrast to RT oxidation, where a small amount of HfO oxide could be obtained from the spectra fittings, at the elevated temperature this oxide phase is almost completely diminished.

**Figure 7:**
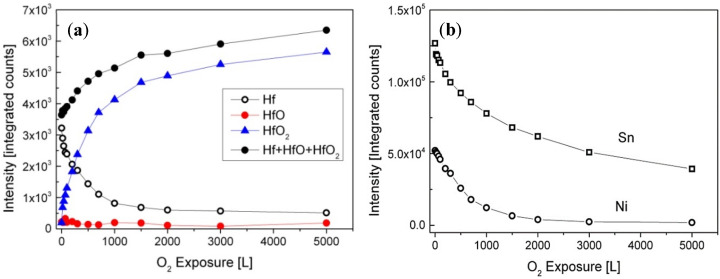
Intensity variation of (**a**) Hf, HfO, HfO_2_, and (**b**) Sn and Ni, during oxygen exposure at 1000 K.

The HfO oxide is known to exist at low temperatures and dissolves above 200 °C [24]. Similar to RT, only slight oxidation of the Sn and no oxidation of the Ni can be seen, but these signals attenuate significantly, and the Ni signal almost disappears entirely after 5000 L exposure. The intensity changes of the Hf, Ni, and Sn during oxidation are depicted in Figure 7.

**Figure 8:**
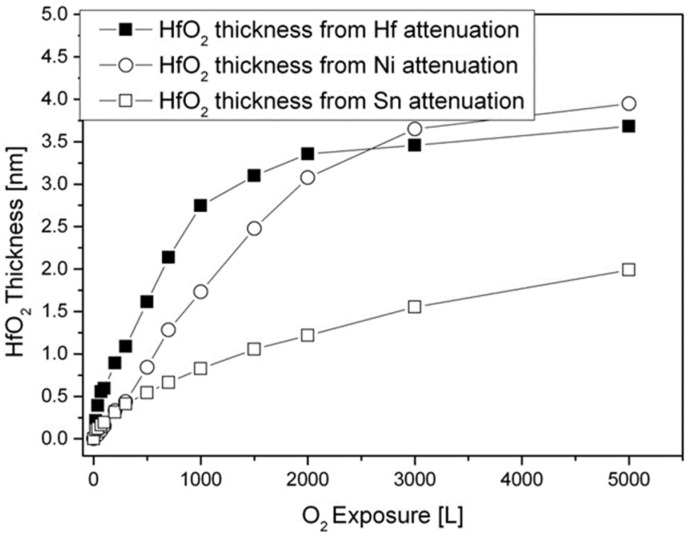
Oxide thickness formed during exposure to O_2_ at 1000 K, as calculated from the attenuation of the Sn, Ni, and Hf peaks.

As in the case of RT oxidation, the oxide thickness and the location where it forms (above or below the Sn layer) can be evaluated and deduced from the attenuation of the Sn, Ni, and Hf signals, and by using Equation (1), as shown in Figure 8. It seems that the oxide thickness calculated from the Hf and Ni attenuation is about twice the thickness calculated from the Sn attenuation. Since a significant Sn signal arrives from the initially Sn-segregated overlayer, the attenuation of the Sn signal is mostly caused by the HfO_2_ formed on top of it, while the attenuation of the Ni and the metallic Hf intensities are governed by both the oxide formed beneath and above the Sn overlayer due to oxygen-induced migration of Hf to the surface. Embracing this relatively simplistic view indicates that the total HfO_2_ oxide formed at 1000 K (after 5000 L exposure) is about 3.5 nm, where a ~2 nm oxide is formed beneath the Sn layer and a ~1.5 nm above it.

### 3.4. The Interaction with H_2_O at RT and 1000 K

**Figure 9:**
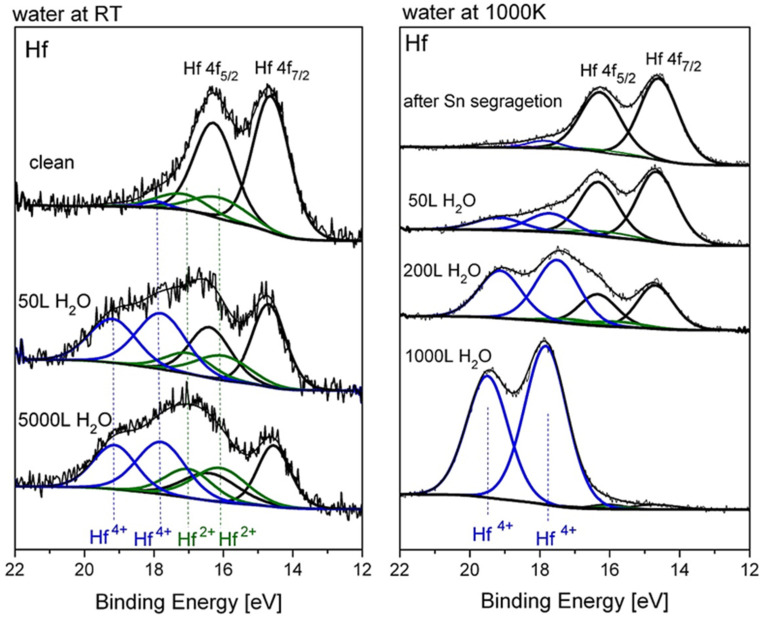
Representative Hf spectra after exposure to H_2_O at RT and 1000 K.

**Figure 10:**
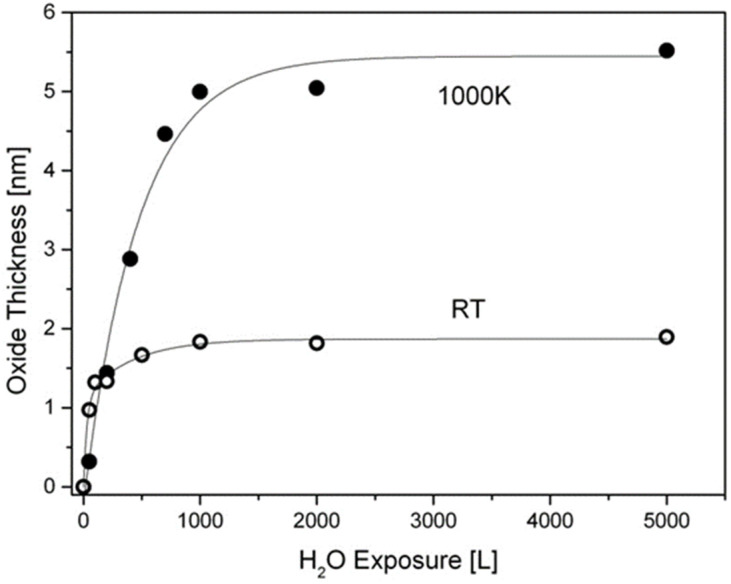
Oxide thickness formed during exposure to water vapor at RT and 1000 K (the lines are a guide for the eye).

Similar to the experiments with oxygen, the exposures to water at RT were performed on the sputtered clean surface, while at 1000 K they were performed after vacuum annealing and Sn segregation. The XPS spectra of Hf after water vapor dosing at RT and 1000 K are depicted in Figure 9. As expected, the oxide film formed at RT contains mainly HfO_2_ in coexistence with some HfO, and it reaches a thickness of up to ~2nm, as calculated from Hf attenuation. At 1000 K HfO_2_ is in fact the only oxide formed, and the metallic Hf signal is almost totally attenuated after 1000 L H_2_O exposure, indicating a faster oxidation rate compared to oxygen. The hafnium oxide thicknesses formed during exposure to water vapor at RT and 1000 K are presented in Figure 10. The Ni and Sn signals were measured only at the beginning and the end of the experiment; they showed attenuation with no signs of Ni oxidation and only a very slight shoulder in the Sn spectrum (similar to Figure 3), indicating some minor SnO formation. Comparing these results to those obtained during oxygen exposure shows that the HfNiSn alloy is more susceptible to water vapor than to oxygen. The preference for oxidation by oxygen or by water vapor can stem from the metal oxidation tendency or from the oxide properties. For example, in contrast to the current work, the oxidation TiNiSn was more severe in oxygen compared to water vapor [10], in agreement with the higher susceptibility of Ti metal to oxygen compared to water vapor [25,26]. On the other hand, Hf oxide was found to be a weak diffusion barrier for water-derived oxidizing species [27], which can explain the thicker oxide formed by water.

### 3.5. Surface Oxidation during System Bakeout

**Figure 11:**
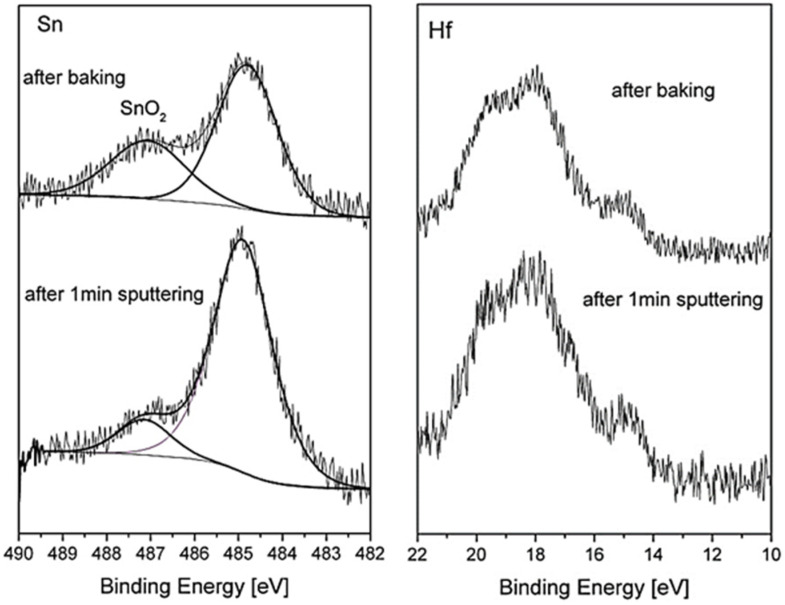
Sn and Hf spectra taken from HfNiSn sample after system bakeout and after a short sputtering of the surface. The SnO_2_ layer is mostly removed while still not noticeably affecting the Hf oxide.

**Figure 12:**
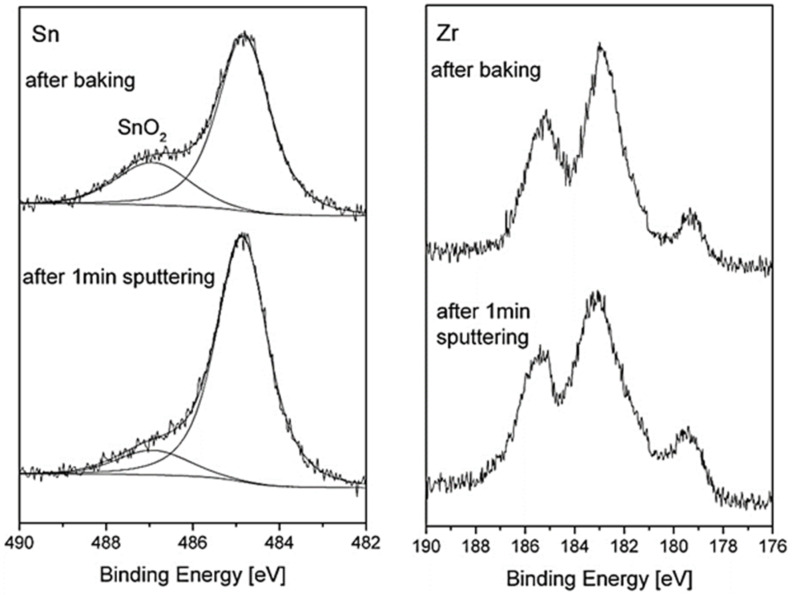
Sn and Zr spectra taken from ZrNiSn after baking and after a short sputtering of the surface. The SnO_2_ layer is mostly removed while still not noticeably affecting the Zr oxide.

While the previous paragraphs described the oxidation behavior under well-controlled conditions, it was found that analyzing the surface after system bakeout (following the loading of the samples into the vacuum chamber) can give additional understanding on the reactions that occur on the surface during heating for an extent duration in moderate temperatures and higher pressure. The system bakeout was for 48 h, in which the temperature on the samples increased to ~420 K, and the pressure changed from ~1 × 10^−5^ to ~5 × 10^−8^ Torr at the end. As expected for a UHV system during bakeout, the atmosphere contained mostly water vapor with some hydrogen and CO traces, as verified with a residual gas analyzer. The XPS spectra taken after the system bakeout reveals Hf oxidation and a pronounced oxide shoulder of the Sn, as shown in Figure 11. It is also notable that the Sn oxide shoulder was shifted to higher binding energy by ~2.1 eV, indicating the formation of Sn^4+^ oxidation state (SnO_2_), compared to a shift of ~1.8 eV that we attribute to SnO. Moreover, short sputtering of the surface removed most of that SnO_2_ oxide, while still not noticeably affecting the Hf oxide, indicating that this SnO_2_ film was formed mainly on top of the oxide layer. Similar results can be also seen for the ZrNiSn sample, as depicted in Figure 12. Further sputtering is expected to remove the remaining SnO_2_ but can also damage the hafnium or the zirconium oxides. These results are also in accordance with the work of Kang et al. [9], who reported the formation of a thin SnO_2_ layer on the surface of a (Hf_0.6_Zr_0.4_) NiSn alloy during long oxidation in air.

## 4. Summary and Conclusions

Comparing the results obtained here to the results found during previous reports on the other two alloys of the HH family, i.e., TiNiSn [10] and ZrNiSn [11], can give an insight into the surface composition and the initial oxidation mechanism. For all three alloys, it was found that during heating in vacuum, Sn segregates to the surface, creating a sub-nanometer overlayer. The driving force for this segregation was derived from the significantly lower surface energy of the Sn compared to the other components. It should be noted that since usually there is a similarity between segregation to free surfaces and to grain boundaries, we should also expect some enrichment of Sn at the grain boundaries, which may affect the transport behavior across the sample or a device.

The initial oxidation of the MNiSn alloys (M = Hf, Zr, Ti), is clearly governed by the component’s oxidation enthalpies of the Hf, Ti, and Zr (and therefore the free energy), which are considerably more negative than that of the Ni or Sn. The initial surface oxidation occurred by extracting some Hf, Ti, or Zr cations from the alloy to the surface by oxygen-induced segregation (and its oxidation on the surface) and by oxygen penetration into the alloy subsurface and oxidation. It was also found that Sn was slightly oxidized, probably to SnO, while no evidence of Ni oxidation was seen. It should be expected that this trend would prevail also for the initial oxidation of more complex alloys based on these components. The HfNiSn alloy is more susceptible to water vapor compared to oxygen, in contrast to TiNiSn, which was found to oxidize preferably in oxygen [10]. In the case of long oxidation and higher pressure (as in the case of the system bakeout), the Sn segregation and oxidation to SnO_2_ on the surface can play a more significant part in the overall oxide composition, as was also demonstrated by Kang et al. [9]. The oxidation tendency of the Hf, as well as Ti and Zr, implies that appropriate sealing of the device is necessary in order to protect it from the atmosphere at elevated temperatures.

As for the oxidation behavior of the alloys during exposure to atmospheric air at elevated temperatures, where thicker oxide layers are expected, one point should also be taken into account, namely the kinetic considerations of the oxide growth and Sn segregation that can greatly influence the local alloy composition near the surface and the overall corrosion of the HH alloys. It is therefore necessary to overcome the pressure gap to obtain a complete understanding of the corrosion behavior under more realistic and technological conditions. These phenomena are explored in the work that follows this study.

## Figures and Tables

**Table 1:** Binding energy (eV) of the elements in the alloys and the known literature values for the pure elements, with chemical shifts shown in brackets.

	Hf 4f_7/2_	Zr 3d_5/2_	Ti 2p_3/2_	Ni 2p_3/2_	Sn 3d_5/2_
Pure elements [12,13,14,15]	14.3	178.9	453.9	852.6	485
ZrNiSn [11]	–	179.1 (+0.2)	–	852.8 (+0.2)	484.6 (−0.4)
TiNiSn [10]	–	–	454.2 (+0.3)	852.9 (+0.3)	484.5 (−0.5)
HfNiSn (this work)	14.4 (+0.1)	–	–	853.1 (+0.4)	484.4 (−0.4)

## Data Availability

The data presented in this study are available on request from the corresponding author.
